# Influence of Sport Practice and Body Weight on Physical Fitness in Schoolchildren Living in the Campania Region

**DOI:** 10.3390/ijerph19127412

**Published:** 2022-06-16

**Authors:** Annamaria Mancini, Domenico Martone, Daniela Vitucci, Adriano Capobianco, Andreina Alfieri, Pasqualina Buono, Stefania Orrù

**Affiliations:** 1 Dipartimento di Scienze Motorie e del Benessere, Università degli Studi di Napoli Parthenope, 80133 Naples, Italy; annamaria.mancini@uniparthenope.it (A.M.); domenico.martone@uniparthenope.it (D.M.); vitucci@ceinge.unina.it (D.V.); adriano.capobianco@studenti.uniparthenope.it (A.C.); andreina.alfieri@uniparthenope.it (A.A.); pasqualina.buono@uniparthenope.it (P.B.); 2 CEINGE Biotecnologia Avanzate “Franco Salvatore”, 80145 Naples, Italy

**Keywords:** Eurofit battery tests, sport practice, overweight/obesity, health-related physical fitness, physical activity

## Abstract

Background: Physical fitness (PF) levels correlate with health hallmarks at all ages. In this study, w aimed to determine the PF level of schoolchildren from the Campania Region (Italy) through health-related PF (HRPF) components, taking into account body weight and sport practice (SP). Methods: PF level was determined in 565 schoolchildren aged 10–13 (11.7 ± 1.0 yrs; m: 353, f: 212) using some of the Eurofit battery tests. Results: 77% children practiced sport, boys more than girls (86% vs. 63%, respectively; *p* < 0.05). Boys performed better than girls (*p* < 0.05) in the Plate Tapping, Standing Broad Jump, Bent-Arm Hang, and 10 × 5 m Shuttle Run tests; girls performed better in the Sit-and-Reach Test (*p* < 0.05). Conclusion: Overweight/obese status negatively affects the muscular strength of lower limbs, even if it progressively improves during growth. SP was revealed to be a determinant in performance only in some PF tests, likely due to the heterogeneous training level among boys and girls who practice sport.

## 1. Introduction

Sedentary lifestyle in childhood is associated with overweight/obesity (Ow/Ob) and increases the risk of non-communicable diseases (NCD), such as cardiovascular and metabolic diseases, in adulthood [1,2]. In the Mediterranean area and, in particular, in Southern Italy (Campania region), emerging data on children’s lifestyle are seriously alarming. According to the “HBSC 2018–2019 Regione Campania” [3] and “Okkio alla salute 2019” [4] surveys, in Campania child overweight, evaluated as body mass index (BMI), refers to about 25% of both 8-/9-year-old and 11–15-year-old children. The same reports indicate that obesity is prevalent among 8-/9-year-old children (18.8%), whereas it decreases to 5.7% in 11–15-year-olds; among the latter, male obesity peaks at 9% and female obesity at 3.6%, suggesting a permanent critical condition, especially in boys. Such a trend is coupled with low physical activity (PA) levels: in fact, about 13% of 11–15-year-old children from the Campania region do not exercise (both recreationally or competitively) during the week, outside of school time, and spend 3–4 h/day or more during the weekend on sedentary activities like watching TV or playing videogames [3,4]. 

Ow/Ob along with an inadequate PA leads to a poor physical fitness (PF) in youth; as a consequence, such a condition negatively affects PF levels in adulthood, determining higher cardiovascular disease (CVD) risk and general poor health [5,6,7,8,9,10,11,12,13,14,15]. In particular, it has been observed that PA, PF, skinfold, and waist circumference are all independently associated with clustered CVD risk, such as systolic blood pressure, triglycerides, total cholesterol/HDL ratio, and HOMA score, in children aged 9 and 15 year old [5]. Conversely, it has been well demonstrated that adequate PA levels (60 min per day) in children positively correlate to muscle, bone, and mental health, and positively affect cognitive and psychosocial functions [16,17]. Therefore, given the connections, the analysis of the body weight (BW) and PA and PF levels in childhood is relevant to preventing not only fitness deficiencies, but also obesity and diseases in adults.

PF can be assessed through specific motor skill indicators (muscular strength, speed, agility, balance, coordination, flexibility, and cardiorespiratory fitness) widely recognized as health-related PF (HRPF) components. All of them can be evaluated by means of simple, standardized, and reliable physical tests, such as the Eurofit battery tests, developed for school-age children and applied since 1988 [18,19,20].

The Eurofit battery consists of anthropometric measurements of body weight and height and nine physical tests, including the Flamingo Balance Test, Plate Tapping, Sit-and-Reach Test, Standing Broad Jump Test, Handgrip Test, Sit-Ups in 30 s Test, Bent-Arm Hang Test, 10 × 5 m Shuttle Run Test, and 20 m Shuttle Run Test [18]. The success of this battery relies on its field-based utility and its inexpensive and simple mode of application, with minimal equipment requirements [21]. Each individual test has an excellent test–retest reliability [22,23,24,25] and many of them have been applied to children and adolescents in several international projects, such as the AVENA and the HELENA studies and others [26,27,28,29,30,31,32,33].

Recently Tomkinson et al. (2017) provided sex- and age-specific European normative values for children and adolescents (aged 9–17) in order to make Eurofit data comparable by analyzing more than 2.5 million performances extracted from 98 studies from 30 European countries [34].

However, PF levels in children depend on several determinants, including socio-cultural and economic factors, and others related to lifestyle, which can vary widely among different states and even within the same country/region [35,36,37]. In Italy, a few studies have been carried out since the 1990s in some specific regions [26,27,28,29], but, to the best of our knowledge, no data are available for the Campania region up to now.

Based on this scenario, the main objectives of this pilot study are (1) to assess the sex- and age-specific HRPF components in a random cohort of young boys and girls aged 10–13 living in the Campania region by means of some of the Eurofit battery tests [18]; (2) to analyze the associations between HRPF components, BW, and sport practice (SP); (3) to evaluate BMI and SP as potential performance determinants of HRPF scores; and (4) to compare the obtained results with known age- and sex-matched Eurofit normative values [34]. Based on the surveys quoted above, we hypothesize that the Ow/Ob rate may negatively affect PF, whereas SP could act as a positive modulator of health-related motor skills.

## 2. Materials and Methods

### 2.1. Study Design

Our cohort was selected by convenience from secondary schools of both rural and urban areas of the Campania region (Italy). The activities were carried out at school, during the curricular hours of physical education (PE), in collaboration with PE teachers. SP and anthropometric assessment were evaluated on the first meeting. A participant was considered an athletic child if engaged in a sport activity outside school hours, training in 2–3 sessions/week for 1–1.5 h/session. As for the weight status, participants were grouped in normal weight or Ow/Ob using the national age and gender-specific cutoff points for BMI [38]. On the test day, children performed a light warm-up (10 min brisk walking and free bodyweight exercises), followed by the test assessment. Each test was fully explained to all participants beforehand and supervised by skilled specialists in movement sciences in order to minimize the potential intra- and inter-rater variability.

### 2.2. Participants

A random sample of 565 children was recruited in Campania schools. The sample was composed of 353 boys and 212 girls (aged 11.7 ± 1.0 and 11.6 ± 1.0 years old, respectively; Table 1). Children with medical issues or physical or mental disabilities were not included in the study. In our cohort, 77% exercised at gyms or sports centers (86% boys, 63% girls). The main sports played by the boys were soccer (n.123), basketball (n.109), rowing (n.17), volleyball (n.15), and others (n.38), whereas the girls practiced mostly dancing (n.51), volleyball (n.47), rowing (n.12), and others (n.21).

Parents or legal representatives of the young participants were instructed on the scope of the study, including its potential benefits and risks, and then asked to sign an informed consent, stating also that each child could be withdrawn from the study at any time or decide to not perform a specific item included in the PF evaluation protocol (approval code by the Ethical Committee of University of Naples “Federico II”: n. 376/19). Each child was assigned an identification number to ensure anonymity. The study was conducted according to the Declaration of Helsinki (2013) and the European Union recommendations for Good Clinical Practice [39].

### 2.3. Anthropometric Measures

Body weight and height were measured to the nearest 0.1 kg and 0.1 cm, respectively, with children in bare feet and light clothing using standardized equipment. BMI was calculated as weight in kilograms divided by the square of height in meters (kg·m^−2^). According to this, we considered 227 normal weight (BMI: 20.0 ± 2.0 kg·m^−2^) and 126 Ow/Ob boys (BMI: 24.3 ± 2.2 kg·m^−2^); moreover, we considered 181 normal weight (BMI: 19.4 ± 2.5 kg·m^−2^) and 31 Ow/Ob girls (BMI: 27.0 ± 2.4 kg·m^−2^).

### 2.4. Physical Fitness Test

PF was evaluated by using the Eurofit battery tests [18], except for the Handgrip Strength and Sit-Ups tests. All items, except for the 20 m Shuttle Run Test, were conducted twice and the best performance was recorded.

*Flamingo Balance*. This test measures the ability to balance successfully on a single leg [18]. Children were asked to stand barefoot with the preferred leg on a little beam while the free leg was flexed at the knee joint and held at the ankle joint close to the buttocks for one minute. The number of falls/min was recorded for analysis. The test was failed if there were more than 15 falls in the first 30 s.

*Plate Tapping*. This test measures the speed and the coordination of upper limb movement [18]. Two discs were placed with their centers 60 cm apart on a table with a rectangular plate equidistant between both discs. Children were asked, standing in front of the table with feet slightly apart and the non-preferred hand on the rectangle, to move the preferred hand back and forth between the discs over the hand in the middle as quickly as possible for 25 full cycles (50 taps). The best time, expressed in seconds and tenths of seconds, was recorded.

*Sit-and-Reach*. This test assesses the flexibility of the lower back and hamstring muscles. It is performed sitting with the legs straight and barefoot soles placed flat against a standard box with a centimeter scale on the top [18]. The young participants were asked to bend the trunk and reach toward the centimeter scale as far as possible. The distance reached in centimeters on the scale was recorded.

*Standing Broad Jump*. This test assesses lower-limb explosive strength [18]. From a starting line, with feet roughly shoulder-width apart, children were asked to jump as far as possible, landing on both feet without falling backwards (it was not allowed to put hands on the floor). The best result, measured in centimeters, was recorded.

*Bent-Arm Hang*. This test assesses upper-limb relative strength and endurance [18]. Each participant was assisted to grasp the horizontal bar at a specific height, so that the chin was level with it, and asked to keep the suspended position as long as possible. The best time, expressed in seconds and tenths of seconds, was recorded.

*10 × 5 m Shuttle Run*. This test measures speed and agility [18]. Each shuttle was considered complete only if both feet had fully crossed the line. The total time to complete the task was measured.

*20 m Shuttle Run*. This test, also called the Leger test, measures maximal running aerobic fitness [18]. According to the standard procedure, children were asked to run continuously between two lines 20 m apart, in time to recorded beeps. The test ended after the second warning when the participant was not able to keep the rhythm. The number of stages was recorded and used for analysis.

VO_2_max was obtained by introducing the age (A) and the final speed (S = 8 + 0.5 × last stage completed) into the following formula [40]:

VO_2_max = 31.025 + 3.238S − 3.248A + 0.1536SA

The healthy cardiorespiratory fitness (CRF) zone was set as VO_2_max > 42 mL·kg^−1^·min^−1^ for males aged 10–17 years according to international criterion-referenced standards [41].

### 2.5. Statistical Analysis

Data are presented as means and standard deviations (mean ± SD). Comparisons between groups were determined with the Student’s *t*-test for all normally distributed variables or with a non-parametric test (Mann–Whitney test) for continuous variables (BMI, Flamingo Balance, Plate Tapping, Bent-Arm Hang for both boys and girls, Sit-and-Reach only for boys). For the dichotomous variables, the Chi-square test was used to evaluate the statistical significance between different weight categories (normal vs. Ow/Ob) and SP (no vs. yes). Multiple linear regression was used to model the relationship between BMI and SP (no vs. yes) and each HRPF component score. The 50th percentile value by gender and age for each HRPF component was determined using the LMS method [42]. Data were imported into the LmsChartMaker software v.2.54 (by Tim Cole and Huiqi Pan) and the curves corresponding to 50th percentile were obtained by smoothing three age-specific curves: L (lambda; skewness), M (Mu; median), and S (sigma; coefficient of variation). Jamovi software (version 2.2) was used for the analyses [43]. The significance level was set at *p* < 0.05.

## 3. Results

Table 1 provides the anthropometric data, the percentage engagement in SP, and the average values of the HRPF components of participants (boys and girls).

Boys compared to girls had significantly greater height and weight (*p* < 0.05). Almost one child out of three (27.8%) was Ow/Ob, with boys significantly outnumbering girls (35.8% vs. 14.5%, respectively; *p* < 0.05). Most of the recruited children practiced sports (77%), but there were many more athletic boys than girls (86% vs. 63%, respectively; *p* < 0.05). Boys performed significantly (*p* < 0.05) better than girls in most of the Eurofit tests: Plate Tapping, Standing Broad Jump, Bent-Arm Hang, and 10 × 5 m Shuttle Run. On the other hand, girls performed significantly better in the Sit-and-Reach Test (*p* < 0.05). No significant differences were found in the Flamingo Balance test scores between genders. Very few girls performed the 20 m Shuttle Run Test, so the corresponding results are not reported. The effects of SP (no vs. yes) and weight status (normal vs. Ow/Ob) on different HRPF components in boys and girls are shown in Table 2.

By analyzing the results in relation to SP, athletic boys performed better than sex-matched sedentary participants in five out of seven tests: Flamingo Balance (*p* < 0.001), Plate Tapping (*p* = 0.001), Sit-and-Reach (*p* < 0.001), Standing Broad Jump (*p* < 0.001), and 20 m Shuttle Run (*p* = 0.013) tests. As for girls, SP yielded better results only in the Flamingo Balance (*p* < 0.001) and in the Sit-and-Reach (*p* < 0.001) tests.

As for the weight status, normal-weight (NW) boys performed significantly better than their Ow/Ob counterparts in the Standing Broad Jump (*p* < 0.001) and in the 20 m Shuttle Run (*p* = 0.042) tests. On the other hand, NW girls achieved better results in the Plate Tapping (*p* = 0.036) and Standing Broad Jump (*p* < 0.001) tests and in the 10 × 5 m Shuttle Run Test (*p* = 0.003) compared to their Ow/Ob counterparts.

Among the Eurofit tests performed by our cohort, only the Standing Broad Jump and the 20 m Shuttle Run tests, carried out by the boys, were both sensitive to weight status and to SP. Consequently, a multiple linear regression model was applied in order to quantitatively evaluate the effect of both variables on the performance (Table 3 and Table 4). We found that both BMI and SP, regardless of age, affected the performance to a very low degree.

Data obtained in the 20 m Shuttle Run Test allowed the CRF to be estimated in the male sample; we found that more than 25% of boys showed a VO_2_max lower than 42 mL·kg^−1^ min^−1^, which is considered the healthy fitness zone threshold [41].

Despite the nature of this pilot study, in which the sample size is not representative of the population of 10–13-year-old children in the Campania region, the 50th percentile for each HRPF component by gender and age was tentatively determined (Table 5) to allow a comparison with European normative values [34].

The data reveal that, while growing up from 10 to 13 years old, both boys and girls tended to improve their PF, with a few exceptions. Boys showed the best enhancement in the Bent-Arm Hang (>200%) and in the 20 m Shuttle Run tests (+108%); on the other hand, their performance was almost stable in the Flamingo Balance Test (+3%), whereas it decreased slightly in the Plate Tapping test (−11%). As for the girls, the best improvements were recorded in the Sit-and-Reach (+51%) and in the Bent-Arm Hang (+50%) tests, although, in the latter case, the absolute recorded time was still too short. Finally, like the boys, 13 and 10 year-old girls performed the Flamingo Balance Test in a similar way.

## 4. Discussion

Nowadays children and adolescents around the world spend more and more time in a sitting, reclining, or lying position, with an energy expenditure of ≤1.5 metabolic equivalents (METSs) [44]. A recent survey stated that 81% of adolescents aged 11–17 do not reach the minimal level of PA required at their age, with significant differences across genders, regions, and countries [45].

Campania is one of the Italian regions with the lowest per capita income; in fact, according to the most recent survey by the Istituto Nazionale di Statistica, in Campania the share of households in relative poverty is higher (24.9%) compared to the national share (11.8%) [46]. Moreover, the Campania region provides the most limited public spaces available to exercise safely in free time and the highest number of schools without a proper gym for students (>70%) [47]; on the other hand, it is acknowledged that the more developed and higher-income districts may provide more educational resources and public spaces for children so they have more opportunities to participate in PA [48].

The present study reports, for the first time, the assessment of PF in a random cohort of schoolchildren living in the Campania region (Italy), evaluated by using some of the Eurofit battery tests. Our results show a gender-based difference in almost all the proposed tests; boys performed statistically better than girls in the Plate Tapping, Standing Broad Jump, Bent-Arm Hang, and 10 × 5 m Shuttle Run tests, but not in the Sit-and-Reach Test. Such results are not surprising; in fact, biological maturation and age exert a decisive influence on physical performance in boys and girls [49,50,51,52]. Although there are no differences in the pre-puberty stages [53,54], they arise when reaching peak speed-to-height (PHV; 9–15 years in girls, 12–16 years in boys) [50]. Both males and females in our cohort had an average age of 11.5 years, revealing that they were in the critical phase of their development, especially the girls. At this age, some motor skills can be slightly impaired due to the growth spurt characterized by a rapid acceleration phase, usually lasting one year, such as balance [55]. In agreement with this well-documented knowledge, the boys and girls of our cohort showed similar performance in the Flamingo Balance Test. Such a finding was also observed by Milanese et al. in a cohort of 193 children (128 boys) aged 6–12 [56]. Nevertheless, when SP was considered, both athletic boys and girls of our cohort showed better postural control than their sedentary counterparts (16.9 ± 4.4 vs. 11.7 ± 4.4 falls/min, *p* < 0.001 for boys; 20.5 ± 5.3 falls/min vs. 11.4 ± 4.8 falls/min, *p* < 0.001 for girls), confirming that a constant SP, characterized by hundreds of short activities challenging balance, allow athletic children to acquire better postural control ability [57].

As for the Bent-Arm Hang and 10 × 5 m Shuttle Run tests, the scores recorded among athletic and non-athletic boys were not different. In the first case, it can be hypothesized that the evaluation of the static muscle strength of the upper limbs could not be a proper indicator to detect potential muscle strength improvements related to the most practiced sports within our male cohort. In fact, elements such as swings, throws, and kicks, present in volleyball, basketball, and soccer, contribute mainly to the improvement of the dynamic muscle strength of the upper and lower limbs [58,59,60]. On the other hand, when agility is taken into consideration, two putative concomitant factors could explain the observed phenomenon: the heterogeneous training level among athletic boys and the common maturation stage of all male participants [61]. In this context, Zemková et al. [62] demonstrated that the improvement in agility performance in more experienced basketball players was greater compared to their less experienced counterparts. Perhaps a significant difference in the agility test could have been found if we had compared more experienced athletic boys to others; although this goal was out of the scope of this study, it could be an interesting issue to explore in the future.

Results from the present pilot study are in agreement with the “Okkio alla salute” and “HBSC 2018–2019 Regione Campania” surveys [3,4], highlighting that, although 77% of our cohort (86% boys; 63% girls) were engaged in regular SP, 27.8% were Ow/Ob. This condition negatively affects some HRPF components, like cardiorespiratory endurance and upper limb muscular strength, in agreement with previous studies, and confirms the relationship among PA levels, BMI, and PF [27,28,63,64,65,66]. Negative effects due to Ow/Ob have also been reported in both sexes for the Standing Broad Jump Test, and in girls for the 10 × 5 m Shuttle Run Test. Nevertheless, boys performed better than girls in muscular strength tests of the upper and lower limbs and in speed–agility, whereas girls performed better in the flexibility test.

It is widely known that a higher BMI determines a decrease in sport skills and an overall lower score of HRPF components in children [63,64,65,66,67,68,69,70,71,72]. These effects are generally explained by both environmental and biological factors, such as SP, motivation, peer influence, and body composition [3,4,63]. It has been demonstrated that Ow/Ob children have a disadvantage performing in weight-bearing activities, such as shuttle and jump tests, due also to a reduced motivation to provide maximal effort during strength tests [64,65,66]. As a matter of fact, the motor skills that require the projection of the body through space, such as running, jumping, and supporting the body off the ground, are often impeded by the presence of excessive body weight. Although we did not assess body composition in the present study, the improvements of performance observed with increasing age, mostly in boys, suggest an increase in free fat mass and a reduction in fat mass, as expected during puberty [73]. Moreover, the increased mechanical work needed to lift the body off the ground in sport activities such as soccer, basketball, dancing, and volleyball may have had a positive influence on muscular fitness. Such environmental and biological conditions might have led to better muscular strength in the boys and girls of our cohort who were engaged in SP outside of school time. Accordingly, we observed that our cohort performed better in the Eurofit tests whose skills mirrored the practiced sport. Hence, young soccer players had better results in the 20 m Shuttle Run Test, basketball or volleyball players in the Standing Broad Jump Test, and dancers in the Flamingo Balance and Sit-and-Reach tests. In particular, our male cohort practiced mostly basketball and soccer, and, as a consequence, performed better in the Standing Broad Jump and in the 20 m Shuttle Run tests, both sensitive to SP and BMI. Nevertheless, these two factors were not sufficient to explain the results obtained among boys, reinforcing the instrumental role of pubertal development [74]. Hence, during puberty, it is paramount to develop all the basic movement skills, not only some sport-specific abilities, and our data demonstrate that, when this task is not pursued, SP alone does not guarantee good performances in the Eurofit tests not reflecting the motor abilities of the practiced discipline [61]. Therefore, the primary achievement for children’s trainers should be to stimulate young players to engage in multilateral activities in order to let their motor skills fully develop [75].

**Figure 1:**
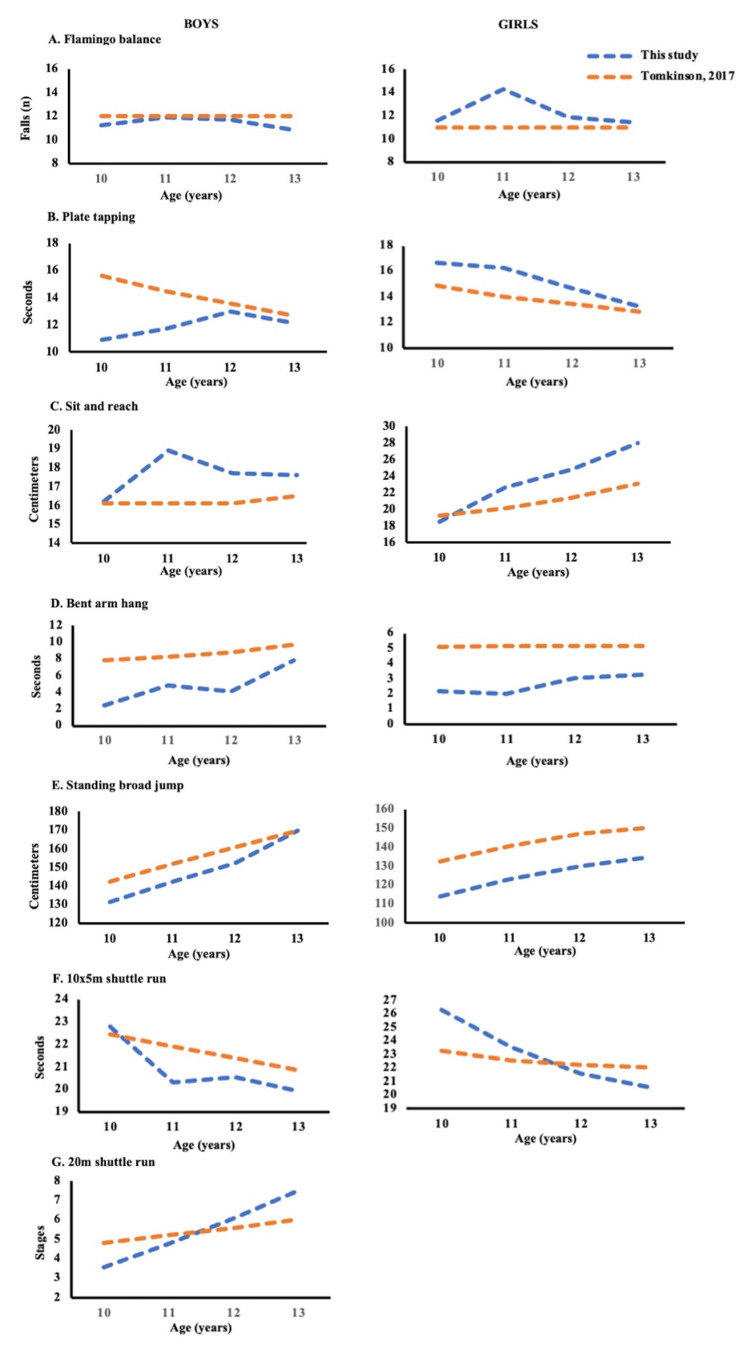
Comparison of 50th-percentile values of HRPF components evaluated in this study with the average score by gender and age obtained from more than 90 European studies [34].

The comparison between the 50th-percentile values related to each specific HRPF component evaluated in this study and sex- and age-matched Eurofit normative values from 2.5 millions of tests, collected among more than 90 European studies [34], allowed the PF level of our young participants to be analyzed (Figure 1).

The balance performance of boys and girls from the Campania region did not change significantly from 10 to 13 years old, and each average score by sex and age is in agreement with other European studies, except for the worst outcome recorded in 11-year-old girls (Figure 1A). A different trend was recorded for the data related to speed and coordination of the upper limbs: 12- and 13-year-old children’s performance was better aligned to other studies [34] than the scores of younger children (Figure 1B). As for flexibility, we observe a similar ability between our cohort at age 10 (boys and girls) and the age-matched European children; moving from 11 to 13 years old, the recorded measurements for males and females were higher compared to the average scores reported in the other studies (Figure 1C). On the contrary, the poorest performances were obtained in the Bent-Arm Hang and in the Standing Broad Jump tests (Figure 1D–E); although these specific HRPF components showed a growth-dependent improvement, our cohort never matched the corresponding reference values (by age and sex), except for 13-years-old boys in the Standing Broad Jump Test [34]. Finally, data from the two different shuttle run tests improve from the youngest participants, with performances below the 50th percentile, to the oldest, with performances above the 50th percentile, compared to the corresponding reference values [34]. Although our sample is not representative of the whole population of 10–13-year-old children from the Campania region, the PF evaluation of our random cohort reveals that male performances on balance, speed and coordination of the upper limbs, and agility were better than the European collected data reported by Tomkinson et al. [34]; conversely, males showed lesser skill in flexibility, upper-limb relative strength and endurance, and lower-limb explosive strength. Upon analyzing the female performance, girls had higher flexibility compared to their age-matched European counterparts, whereas they displayed poor balance, speed and coordination of the upper limbs, upper limb relative strength and endurance, and lower-limb explosive strength. Age-dependent behavior was then recorded for the CRF in boys and agility in girls of our cohort, showing in both cases that the performances of 13-year-old children exceeded that of their age- and sex-matched European peers [34].

Taking into consideration the CRF, we found that 80% of the 12- and 13-year-old boys in our cohort had a VO_2_max higher than 42 mL·kg^−1^·min^−1^, the threshold set for the healthy fitness zone [41]; this percentage decreased to 68% in children at 10 and 11 years old, indicating that only one in three younger male participants met the healthy CRF standards. Cardiorespiratory fitness testing has long been considered not only a marker of performance, but also a health-related indicator for the risk of CVD [41,76]. Poor cardiorespiratory fitness in childhood and adolescence is associated also with skeletal and mental health [77,78], and with metabolic syndrome [79], arterial stiffness [80,81], and myocardial infarction in adulthood [82]. This specific test has been used worldwide to test a huge number of children and adolescents because it has a good validity [22] and reliability [23], and it is feasible and safe [83]. Several European studies aimed at defining the fitness cut points to avoid CVD risk, and it is nowadays well known that a red flag is raised if boys aged 8–17 perform below 12 METs (42 mL/kg/min) and girls below 10 METs (35 mL/kg/min). Such a result is reached when a boy runs at 11 km/hour (stage 6) in the 20 m Shuttle Run Test and a girl runs at 9.5 km/h (stage 3) [41]. Hallal et al. [84] showed that more than 80% of children aged 11–13 from 39 countries did not meet the recommended levels of PA. A significant contribution to this decline in PA was due to the long time spent on screen use (such as television, computers, and video games) [85,86,87], determining not only a reduced CRF but also insufficient muscular fitness [10,11,88]. The outcomes obtained among boys in the 20 m Shuttle Run Test in this pilot study suggest that, although 86% of our male cohort was engaged in SP (usually 2 or 3 times/wk), they spent most of their remaining free time in sedentary activities during the week, thus determining an overall inadequate PF level. In addition, we cannot rule out that the percentage of children not reaching the CRF standard levels may be higher, as the VO_2_max assessment was not determined among girls due to the scarce adherence to the specific test. This scenario might imply an increased risk of noncommunicable diseases (such as CVD, diabetes, and obesity) in adulthood [1,2].

The PF levels found in this pilot study enforce the known relationship among regional socioeconomic level, PA, and PF [3,4,48].

In particular, living in a geographic area with the lowest national per capita income, with very few public spaces available for active leisure time and where fewer than three out of 10 public schools have an adequate gym for students [47], results in a specific environmental condition not favoring the promotion of a healthy and active lifestyle. The low PF levels assessed in this pilot study, especially related to the muscular strength in upper and lower limbs of our male and female cohort, are another strong signal of the urgency of planning preventive actions aimed at children and adolescents to guarantee their future health.

A limitation of this study is the small random convenience sample of participants, which is not representative of all school-aged boys and girls (aged 10–13) living in the Campania region. Data collected here are therefore a useful starting point for a larger project in which a representative sample will be taken to gain a better picture of PF levels among children in the Campania region.

## 5. Conclusions

The present pilot study quantitatively describes the levels of PF using some of the Eurofit battery tests in relation with body weight and SP in a random cohort of school-aged boys and girls (aged 10–13) living in the Campania region (Italy); moreover, we compared the 50th percentile value of each HRPF component by age and sex with other European data [34]. Our results show that the standard European PF levels were not reached for every HRPF component; in particular, the muscular strength of the upper and lower limbs was poor even if it improved during growth. Moreover, more than 30% of 10- and 11-year-old and 20% of 12- and 13-year-old boys showed a CRF below the healthy threshold set at 42 mL·kg^−1^·min^−1^ [41].

To the best of authors’ knowledge, this is the first time that PF levels of a random cohort of school-aged boys and girls (aged 10–13) living in the Campania region were assessed. The collected scores, when compared to other studies, provide a view of the healthy status of the selected population, and confirm an alarming condition among the youngest. In fact, our results underline that the selected population, even if engaged in SP, can be Ow/Ob and have a general sedentary lifestyle, contributing significantly to a decline in PF. For the sake of clarity, this study was performed before the COVID-19 pandemic; as consequence, the results reported here may have been exacerbated by the “lockdown” policies and suspension of physical and recreational activities. So, we strongly believe that such a scenario represents the starting point to plan urgent preventive interventions in the young population with a sedentary lifestyle and at high-risk for NCD. In this regard, schools and sport clubs could represent the ideal setting to promote physical activity and health by encouraging children and adolescents to engage in a more active lifestyle.

## Figures and Tables

**Table 1:** Anthropometric, sport practice, and physical fitness characteristics of children.

Variable (Units)	Total (*n* = 565)	Male (*n* = 353)	Female (*n* = 212)	Mean Dif. (%)
Age (years)	11.7 ± 1.0	11.7 ± 1.0	11.6 ± 1.0	1
Weight (kg)	50.4 ± 11.5	51.9 ± 11.4 *	47.8 ± 11.3	8.2
Height (cm)	153.3 ± 10.2	154.3 ± 10.4 *	151.7 ± 9.6	1.7
BMI (kg/m^2^)	21.2 ± 3.3	21.6 ± 3.0 *	20.6 ± 3.6	4.7
Overweight/obese (%)	27.8	35.8 *	14.5	21.3
Sport practice (%)	77	86 *	63	23
Flamingo Balance (falls/min)	12.2 ± 5.0	11.9 ± 4.5	12.9 ± 5.9	8.5
Plate Tapping (s)	14.3 ± 3.7	13.4 ± 3.4 *	15.6 ± 3.7	15.4
Sit-and-Reach (cm)	20.7 ± 8.5	18.2 ± 6.7	24.5 ± 9.5 *	30.5
Standing Broad Jump (cm)	142.3 ± 28.4	151.5 ± 26.6 *	126.6 ± 24.2	17.5
Bent-Arm Hang (s)	4.9 ± 4.0	6.6 ± 4.7 *	2.9 ± 1.5	75.5
10 × 5 m Shuttle Run (s)	21.5 ± 2.9	20.9 ± 2.6 *	23.1 ± 3.0	10.2
20-m Shuttle Run (stage)	5.8 ± 3.1	6.7 ± 3.1	-	-

All values are means ± SD. * *p*-value < 0.05 refers to boys’ vs. girls’ scores. Mean differences (%) between boys and girls are reported.

**Table 2:** Physical fitness scores of children stratified by sex (boys and girls), sport (no sport/sport) and weight status (normal weight and overweight/obese).

BOYS												
Variable (Units)	No Sport	Sport	*p*	Effect	95% CI		Normal Weight	Overweight/Obese	*p*	Effect	95% CI	
	(*n*:51)	(*n*:302)		Size	Lower	Upper	(*n*:227)	(*n*:126)		Size	Lower	Upper
**FB (falls/min)**	16.9 ± 4.4	11.7 ± 4.4	<0.001	1.396	0.463	2.239	12.0 ± 4.5	11.9 ± 4.3	NS	//	//	//
**PT (s)**	17.1 ± 3.8	13.2 ± 3.3	0.001	1.335	0.251	1.975	13.4 ± 3.7	13.6 ± 2.0	NS	//	//	//
**SiR (cm)**	12.3 ± 5.4	18.5 ± 6.6	0.008	−0.83	−1.519	−0.111	17.9 ± 6.2	17.8 ± 6.7	NS	//	//	//
**SBJ (cm)**	135.3 ± 38.6	154.0 ± 25.4	<0.001	−0.725	−1.056	−0.387	157.4 ± 25.0	140.6 ± 26.0	<0.001	0.585	0.359	0.81
**BAH (s)**	4.5 ± 2.7	7.0 ± 4.8	NS	//	//	//	7.2 ± 4.6	5.5 ± 4.5	NS	//	//	//
**10 × 5 m (s)**	21.2 ± 1.9	20.9 ± 2.6	NS	//	//	//	20.8 ± 2.7	21.1 ± 2.4	NS	//	//	//
**20 m (stage)**	3.6 ± 0.1	6.8 ± 3.1	0.013	−1.301	−2.667	0.152	6.9 ± 3.3	6.1 ± 2.4	0.042	0.277	−0.188	0.742
**GIRLS**												
**Variable (Units)**	**No Sport**	**Sport**	***p***	**Effect**	**95% CI**		**Normal Weight**	**Overweight/Obese**	***p***	**Effect**	**95% CI**	
	**(*n*:81)**	**(*n*:131)**		**Size**	**Lower**	**Upper**	**(*n*:181)**	**(*n*:31)**		**Size**	**Lower**	**Upper**
**FB (falls/min)**	20.5 ± 5.3	11.4 ± 4.8	<0.001	1.837	1.051	2.6	12.6 ± 5.9	14.2 ± 6.0	NS	//	//	//
**PT (s)**	15.7 ± 3.3	15.6 ± 3.8	NS	//	//	//	15.4 ± 3.5	16.9 ± 4.0	0.036	−0.458	−0.884	0.028
**SiR (cm)**	20.7 ± 8.4	25.7 ± 9.6	0.01	−0.48	−0.855	−0.099	24.8 ± 9.2	22.9 ± 11.1	NS	//	//	//
**SBJ (cm)**	124.2 ± 22.0	127.9 ± 25.3	NS	//	//	//	128.9 ± 22.8	111.6 ± 27.7	<0.001	0.813	0.398	1.225
**BAH (s)**	2.7 ± 1.4	3.0 ± 1.5	NS	//	//	//	2.9 ± 1.5	2.2 ± 0.5	NS	//	//	//
**10 × 5 m (s)**	//	23.1 ± 3.0	//	//	//	//	22.7 ± 2.8	25.5 ± 2.9	0.003	−1.119	−1.846	−0.382

FB, Flamingo Balance; PT, Plate Tapping; SiR, Sit-and-Reach; SBJ, Standing Broad Jump; BAH, Bent-Arm Hang; 10 × 5 m, 10 × 5 m Shuttle Run; 20 m, 20 m Shuttle Run.

**Table 3:** Determinants of Standing Broad Jump performance in boys according to multiple linear regression analysis.

R = 0.274	R^2^ = 0.075	Adjusted R^2^ = 0.070	F = 14.0	df1 = 2	df2 = 344	*p* < 0.001	
						95% Confidence Interval	
Predictor	b	SE	*t*	*p*	Stand. b	Lower	Upper
Intercept	158.63	11.019	14.4	0.018			
BMI	−1.11	0.47	−2.36	0.227	−0.123	−0.225	−0.026
Sport practice:							
Yes—No	17.3	3.861	4.48	<0.001	0.669	0.375	0.963

R, coefficient correlation; R2, coefficient correlation squared; Adjusted R2, adjusted coefficient squared; standard error; F, F statistic; df1, degree of freedom 1; df2, degree of freedom 2; β, beta coefficient; SE, standard error; *t*, *t* value; Stand. β, standardized beta.

**Table 4:** Determinants of 20-m Shuttle Run performance in boys according to multiple linear regression analysis.

R = 0.299	R^2^ = 0.090	Adjusted R^2^ = 0.070	F = 3.98	df1 = 2	df2 = 81	*p* = 0.022	
						95% Confidence Interval	
Predictor	b	SE	*t*	*p*	Stand. b	Lower	Upper
Intercept	5.74	2.38	2.41	0.018			
BMI	−0.11	0.091	−1.22	0.227	−0.13	−0.341	0.082
Sport practice:							
Yes—No	3.11	1.283	2.42	0.018	1.205	0.216	2.193

R, coefficient of correlation; R2, coefficient of correlation squared; Adjusted R2, adjusted coefficient squared; standard error; F, F statistic; df1, degree of freedom 1; df2, degree of freedom 2; β, beta coefficient; SE, standard error; *t*, *t* value; Stand. β, standardized beta.

**Table 5:** The 50th percentile of the physical fitness test stratified by age and sex (boys and girls).

	BOYS				GIRLS			
**Age (years)**	10	11	12	13	10	11	12	13
**FB (falls/min)**	11.2	11.9	11.7	10.8	11.6	14.3	11.9	11.5
**PT (s)**	10.9	11.7	13	12.1	16.7	16.3	14.7	13.3
**SiR (cm)**	16.2	18.9	17.7	17.6	18.5	22.6	24.8	28
**SBJ (cm)**	131.3	142.3	152.1	169.8	113.9	122.9	129.6	134.5
**BAH (s)**	2.5	4.9	4.1	7.9	2.2	2	3.1	3.3
**10 × 5 m (s)**	22.8	20.3	20.6	19.9	26.3	23.6	21.6	20.6
**20 m (stages)**	3.6	4.8	6.1	7.5	//	//	//	//

FB, Flamingo Balance; PT, Plate Tapping; SiR, Sit-and-Reach; SBJ, Standing Broad Jump; BAH, Bent-Arm Hang; 10 × 5 m, 10 × 5 m Shuttle Run; 20 m, 20 m Shuttle Run.
